# Delivery of Hydrogen Sulfide by Ultrasound Targeted Microbubble Destruction Attenuates Myocardial Ischemia-reperfusion Injury

**DOI:** 10.1038/srep30643

**Published:** 2016-07-29

**Authors:** Gangbin Chen, Li Yang, Lintao Zhong, Shelby Kutty, Yuegang Wang, Kai Cui, Jiancheng Xiu, Shiping Cao, Qiaobing Huang, Wangjun Liao, Yulin Liao, Juefei Wu, Wenzhu Zhang, Jianping Bin

**Affiliations:** 1 State Key Laboratory of Organ Failure Research, Department of Cardiology, Nanfang Hospital, Southern Medical University, Guangzhou, P.R. China; 2 Department of Pharmacy, Nanfang Hospital, Southern Medical University, Guangzhou, P.R. China; 3 Division of Cardiology, University of Nebraska College of Medicine, Children’s Hospital & Medical Center, Omaha, Nebraska, USA; 4 Department of Pathophysiology, Southern Medical University, Guangzhou, P.R. China; 5 Department of Oncology, Nanfang Hospital, Southern Medical University, Guangzhou, P.R. China; 6 Department of Cardiology, Panyu Central Hospital, Guangzhou, P.R. China

## Abstract

Hydrogen sulfide (H_2_S) is an attractive agent for myocardial ischemia-reperfusion injury, however, systemic delivery of H_2_S may cause unwanted side effects. Ultrasound targeted microbubble destruction has become a promising tool for organ specific delivery of bioactive substance. We hypothesized that delivery of H_2_S by ultrasound targeted microbubble destruction attenuates myocardial ischemia-reperfusion injury and could avoid unwanted side effects. We prepared microbubbles carrying hydrogen sulfide (hs-MB) with different H_2_S/C_3_F_8_ ratios (4/0, 3/1, 2/2, 1/3, 0/4) and determined the optimal ratio. Release of H_2_S triggered by ultrasound was investigated. The cardioprotective effect of ultrasound targeted hs-MB destruction was investigated in a rodent model of myocardial ischemia-reperfusion injury. The H_2_S/C_3_F_8_ ratio of 2/2 was found to be an optimal ratio to prepare stable hs-MB with higher H_2_S loading capability. Ultrasound targeted hs-MB destruction triggered H_2_S release and increased the concentration of H_2_S in the myocardium and lung. Ultrasound targeted hs-MB destruction limited myocardial infarct size, preserved left ventricular function and had no influence on haemodynamics and respiratory. This cardioprotective effect was associated with alleviation of apoptosis and oxidative stress. Delivery of H_2_S to the myocardium by ultrasound targeted hs-MB destruction attenuates myocardial ischemia-reperfusion injury and may avoid unwanted side effects.

Acute myocardial infarction is a major cause of mortality worldwide^1^. Early and successful myocardial reperfusion with either thrombolytic agents or primary percutaneous coronary intervention is the most effective strategy to reduce infarct size and improve clinical outcome. However, the process of restoring blood flow to the ischemic myocardium can induce myocardial reperfusion injury, which can paradoxically reduce the beneficial effects of myocardial reperfusion^2^. Animal studies suggest that myocardial reperfusion injury accounts for up to 50% of the final infarct size^2^,^3^,^4^. Therefore, interventions to attenuate myocardial ischemia-reperfusion injury (MIR) are urgently needed.

Hydrogen sulfide (H_2_S), which has long been considered a toxic pollutant, has been recognized recently as the third therapeutic gaseous signaling molecule, following nitric oxide and carbon monoxide. Growing evidence indicate that H_2_S is involved in MIR^5^,^6^,^7^,^8^,^9^,^10^,^11^,^12^,^13^. H_2_S is produced by cystathionine-β-synthase (CBS), cystathionine-γ-lyase (CSE/CGL) and 3-mercaptopyruvate sulfurtransferase (3-MST) in mammalian cells. Inhibition of endogenous H_2_S production by knockout CSE significantly increase myocardial infarct size^14^, while cardiac specific CSE overexpression reduced infarct size and improved cardiac function^12^. Additionally, exogenous administration of H_2_S at the time of reperfusion decreased infarct size and preserved left ventricular function in a rodent model of MIR^12^. Similar results were observed in a porcine MIR model^9^,^11^. Mechanisms by which H_2_S exerts its cardioprotective effects may include reduction of cardiomyocyte apoptosis^10^,^11^,^12^, inhibition of oxidant stress^10^, anti-inflammatory responses^5^,^9^ and preservation of mitochondrial structure and function^12^. These findings suggest that exogenous administration of H_2_S could be an attractive treatment for MIR.

H_2_S is currently administered either by gaseous H_2_S or H_2_S donors. Inhalation of gaseous H_2_S is poorly tolerated due to the undesirable odor and its irritation of the respiratory tract even at very low concentration^15^. Inorganic donors of H_2_S, Na_2_S and NaHS, widely used in the field, have the advantage of rapidly increasing H_2_S concentration within seconds. However, the effective concentration of H_2_S may not last long within tissue because of rapid degradation of Na_2_S and NaHS. Other long-acting H_2_S donors such as diallyl trisulfide (DATS) and SG-1002 are under investigation^16^,^17^. It is noteworthy that the sensitivity of organs to H_2_S differs, systemic delivery of H_2_S may cause unwanted side effects, including acute change of blood pressure, central neurotoxicity and respiratory depression^18^,^19^,^20^. Direct delivery of H_2_S to the myocardium may avoid the unwanted side effects.

Ultrasound targeted microbubble destruction (UTMD) is the phenomenon where microbubbles when exposed to ultrasound with high acoustic pressures will oscillate and finally collapse. UTMD is widely used to deliver bioactive substances, including therapeutic gases, drugs, and genes, to desired sites^21^. Delivery of oxygen or nitric oxide, using ultrasound and microbubble has been shown to be feasible and of significant therapeutic benefit. For example, using ultrasound and microbubble loaded with nitric oxide, intramyocardial delivery of nitric oxide enhanced the homing of the mesenchymal stem cells into the infracted myocardium and induced the regional angiogenic response^22^. Delivery of oxygen to hypoxic tumor bed with oxygen-filled microbubble and ultrasound increased reactive oxygen species generation and result in enhanced sonodynamic effect^23^. Similarly, development of microbubble encapsulating H_2_S gas could enable targeted H_2_S delivery with ultrasound exposure, unfortunately, reports on this assumption has not been found yet.

We hypothesized that delivery of H_2_S by UTMD attenuates MIR and may avoid unwanted side effects. In this study, we developed microbubble carrying H_2_S (hs-MB) and investigated the effect of ultrasound exposure on release of H_2_S. We further evaluated pathologic features and myocardial function in a rodent model of MIR with ultrasound targeted hs-MB destruction.

## Results

### Preparation and characterization of hs-MB

**Figure 1:**
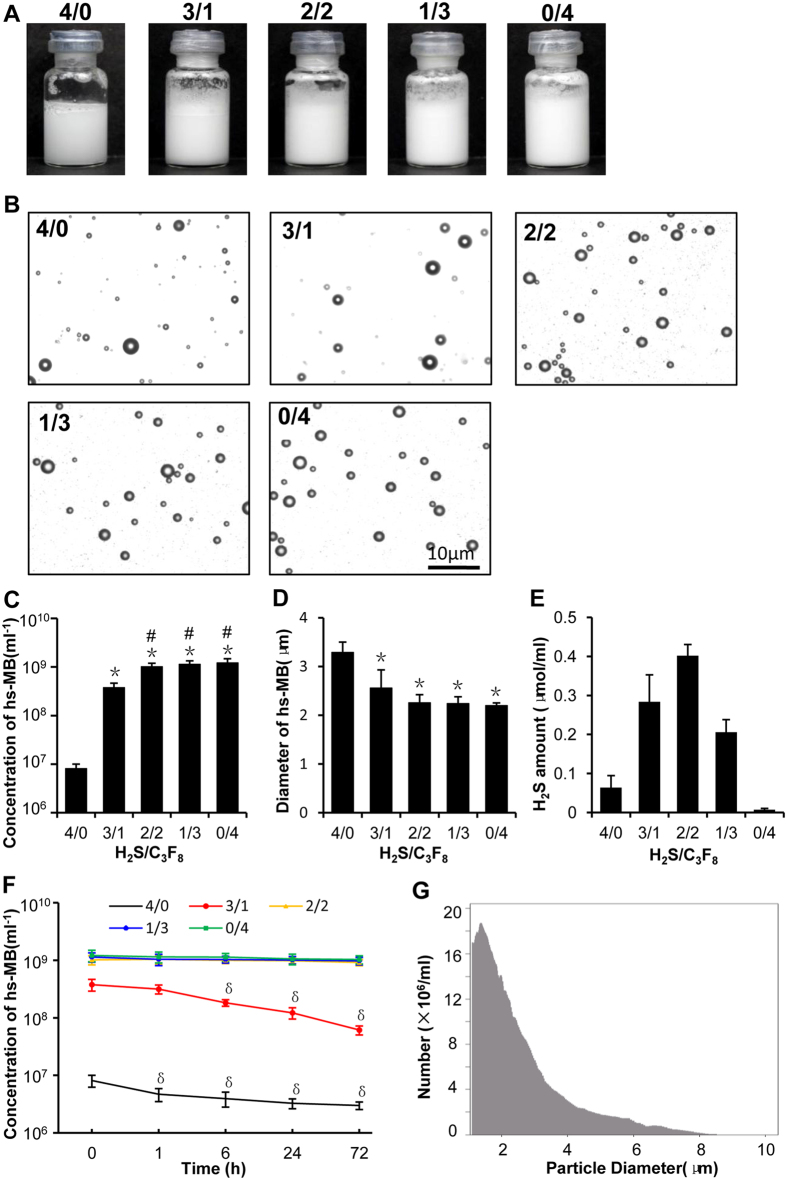
Characterization of hs-MBs prepared with different H_2_S/C_3_F_8_ ratios. (**A**) Appearance of hs-MBs prepared with different H_2_S/C_3_F_8_ ratios. (**B**) hs-MBs under optical microscope. (**C**) Concentration of hs-MBs. (**D**) Mean diameter of hs-MBs. (**E**) Amount of H_2_S encapsulated in hs-MBs. (**F**) Stability of hs-MBs. (**G**) Size distribution of hs-MB prepared with the H_2_S/C_3_F_8_ ratio of 2/2. *P < 0.01, vs H_2_S/C_3_F_8_ ratio of 4/0; ^#^P < 0.01, vs H_2_S/C_3_F_8_ ratio of 3/1; ^δ^P < 0.05, vs baseline (at 0 h). hs-MB indicates microbubble loaded with hydrogen sulfide; H_2_S/C_3_F_8_, volume ratio of hydrogen sulfide and octafluoropropane.

Since H_2_S may efflux from the microbubble shell and resulting in poor stability of hs-MB, we added different amount of octafluoropropane (C_3_F_8_), a large molecule internal gas, to prepare hs-MB for the purpose of achieving stable hs-MB carrying more H_2_S. The hs-MBs were milky in appearance. The hs-MB prepared with H_2_S/C_3_F_8_ ratio of 3/1 was slightly denser in appearance than the one with 4/0 which represented the most the lightest in color. There was no difference in color among hs-MBs prepared with H_2_S/C_3_F_8_ ratio of 2/2, 1/3 and 0/4 (Fig. 1A). The hs-MBs displayed a regular spherical shape without aggregation under microscope (Fig. 1B). The microbubble concentration and size distribution was measured using the Multisizer III Coulter counter. The hs-MB prepared with H_2_S/C_3_F_8_ ratio of 4/0 represented the lowest initial concentration of (8.09 ± 1.88) × 10^6^. At the H_2_S/C_3_F_8_ ratio of 3/1, the initial concentration of hs-MB was increased compared with 4/0 (P < 0.01), while lower than the ratio of 2/2 (P < 0.01). The initial concentrations of hs-MB were not different prepared with H_2_S/C_3_F_8_ ratios of 2/2, 3/1 and 0/4 (Fig. 1C). The hs-MBs prepared with H_2_S/C_3_F_8_ ratios of 3/1, 2/2, 3/1 and 0/4 displayed no difference in diameter (P > 0.05). However, at H_2_S/C_3_F_8_ ratio of 4/0, the diameter of hs-MB was larger than other groups (all P < 0.01) (Fig. 1D). The hs-MB prepared with H_2_S/C_3_F_8_ ratio of 2/2 displayed the highest H_2_S encapsulation of 0.40 ± 0.03 μmol/mL (Fig. 1E).

For stability assessment, the concentrations of hs-MBs were measured at different time points (0 h, 1 h, 6 h, 24 h, 72 h). The concentration of hs-MB prepared with H_2_S/C_3_F_8_ ratio of 4/0 was substantially decreased one hour after preparation (P < 0.05). At the H_2_S/C_3_F_8_ ratio of 3/1, the concentration of hs-MB was reduced 6 hours after preparation (P < 0.05) and kept reducing thereafter. The concentration of hs-MBs prepared with H_2_S/C_3_F_8_ ratio of 2/2, 3/1 and 0/4 were not different in 72 hours (P > 0.05) (Fig. 1F).

To obtain hs-MB carrying more H_2_S and possessing excellent stability simultaneously, the optimal ratio of H_2_S/C_3_F_8_ was figured out to that of 2/2 and was utilized for the following experiment. With this optimal ratio, the hs-MB exhibited a mean microbubble diameter of 2.26 ± 0.17 μm, ranging from 0–8 μm (Fig. 1G) and a concentration of (1.01 ± 0.19) × 10^9^/mL.

### Ultrasound triggered H_2_S release from hs-MB *in vitro*

**Figure 2:**
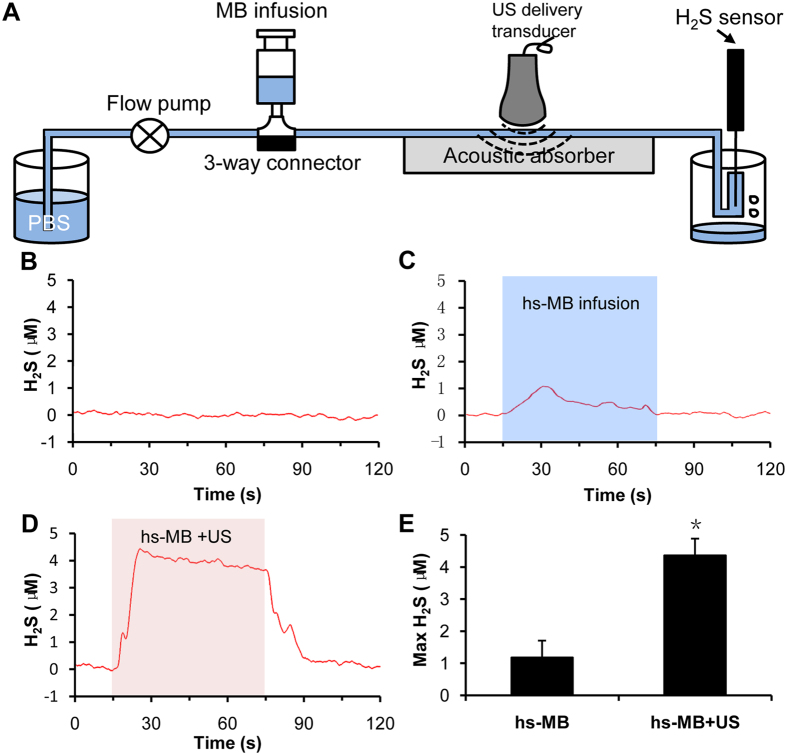
Ultrasound triggered H_2_S release from hs-MB *in vitro*. (**A**) *In vitro* setup of flow system for ultrasound triggered H_2_S release from hs-MB. (**B**) Baseline level of H_2_S. (**C**) Change of H_2_S level during hs-MB infusion. (**D**) Change of H_2_S level during hs-MB infusion and ultrasound irradiation. (**E**) Comparison of maximum concentration of H_2_S. *P < 0.05, vs hs-MB. US indicated ultrasound; hs-MB, microbubble loaded with hydrogen sulfide.

In an *in vitro* flow system, baseline level of H_2_S was fluctuated at 0 μM. During hs-MB infusion, H_2_S level slightly increased. However, the H_2_S level was significantly increased and fluctuated between 4–5 μM during application of ultrasound and hs-MB. The H_2_S level drop back to the baseline when the treatment of ultrasound and hs-MB was stop. The maximum concentration of H_2_S was significantly increased in group treated with hs-MB and ultrasound compared with infusion of hs-MB (P < 0.05, Fig. 2). These results indicated the feasibility of H_2_S released from hs-MB triggered by ultrasound.

### *In vivo* local H_2_S delivery mediated by hs-MB and ultrasound

**Figure 3:**
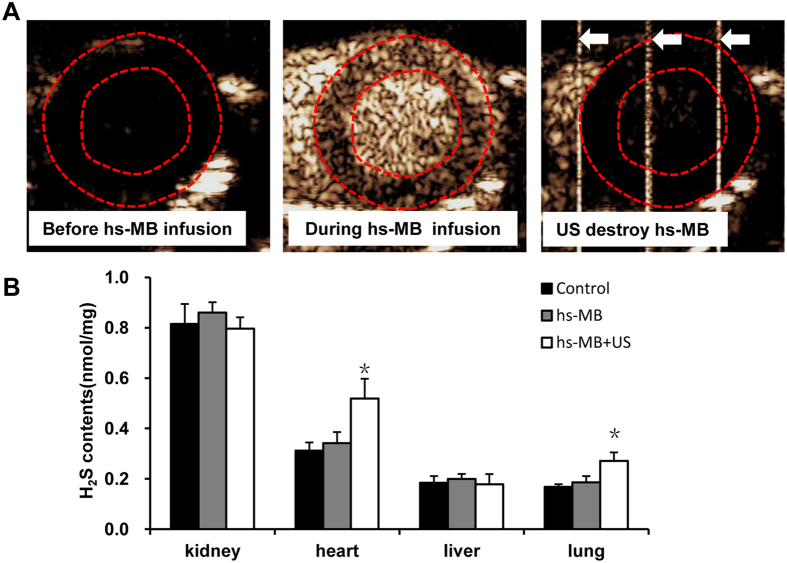
*In vivo* local delivery of H_2_S mediated by hs-MB and US. (**A**) *In vivo* imaging of ultrasound targeted hs-MB destruction in the myocardium. Red dotted line indicated the region of myocardium. White arrow indicated the ultrasound pulse emitted from an ultrasonic cavitation apparatus. (**B**) Comparison of H_2_S concentration in various tissues following treatment. *P < 0.05 vs Control. US indicated ultrasound; hs-MB, microbubble loaded with hydrogen sulfide.

We further examined the local H_2_S delivery mediated by hs-MB and ultrasound in rats. The behavior of hs-MB was evaluated with myocardial contrast echocardiography. No enhanced ultrasound signal in myocardium was observed before hs-MB infusion. After intravenous infusion of hs-MB, the ultrasound signal greatly increased in myocardium. When the ultrasound (1.0 MHz and 1.0 MPa) was turned on, ultrasound signal in the whole myocardium was significantly decreased, suggesting successful fragmentation of hs-MB in the myocardium (Fig. 3A). The heart, lung, liver and kidney were collected for the the H_2_S measurement following treatment. Figure 3B showed that H_2_S in heart was greatly increased in rats treated with hs-MB + US than that of received no treatment (P < 0.05), while there was no difference in rats received hs-MB and no treatment (P > 0.05). Similar result was observed in the rodent lung. However, there was no difference of H_2_S in kidney or liver in three groups (Fig. 3).

### Ultrasound targeted hs-MB destruction limited the extent of MIR

**Figure 4:**
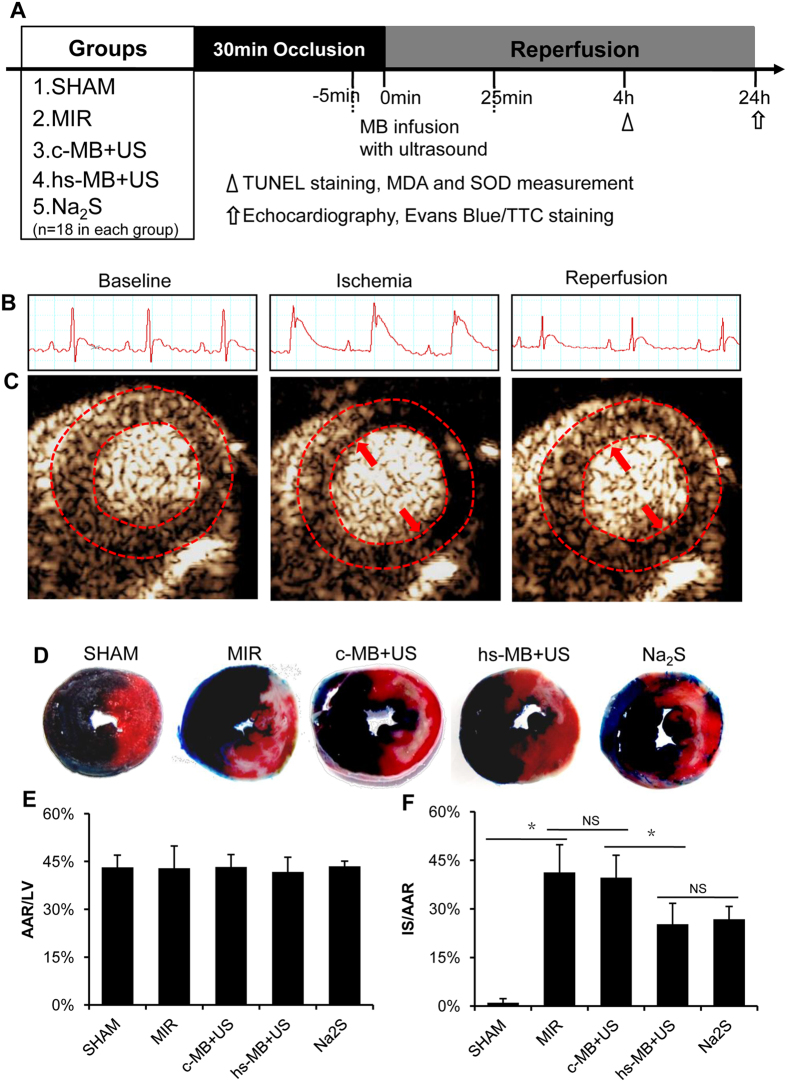
Ultrasound targeted hs-MB destruction limit the extent of MIR. (**A**) *In vivo* experimental protocol showing the groups, intervention and outcomes measurement. (**B**) Electrocardiogram of rats underwent myocardial ischemia and reperfusion. (**C**) Ultrasound imaging of myocardial perfusion during ischemia or reperfusion. Red dotted line indicated the region of myocardium. Red arrows indicated perfusion defect in the anterior wall. (**D**) Representative photographs of mid-ventricular cross sections stained with Evans/TTC. Dark blue stain indicated viable area; White stain indicated infarct region; White plus red stain indicated area at risk. (**E**) Comparison of area at risk per left ventricle (AAR/LV). (**F**) Comparison of area of infarct size normalized to the area at risk (IS/AAR). *P < 0.05. MIR indicates myocardial ischemia-reperfusion injury; c-MB, control microbubble; US, ultrasound; hs-MB, microbubble loaded with hydrogen sulfide; NS, not significant.

To determine the cardioprotective effect of hs-MB + US, a rodent model of MIR was established. Rats were subjected to 30 minutes of LCA ligation followed by reperfusion. ST segment elevation was observed on ECG when LCA was ligated, and partly recovered 2 hours after reperfusion (Fig. 4B). Ultrasound contrasted imaging showed that obvious perfusion defect in the anterior wall (marked by red arrows) was observed when the LCA was ligated. After reperfusion, the perfusion defect in anterior wall partly recovered (Fig. 4C). Myocardial infarction was then evaluated at 24 hours of reperfusion by Evans/TTC dual staining. Representative photographs of mid-ventricular cross sections stained with Evans/TTC are shown in Fig. 4D. The AAR/LV was similar in all of the groups (P > 0.05, Fig. 4E). Compared with SHAM group, MIR caused a significant increase in infarct size (1.0 ± 1.2% vs 41.23 ± 8.57%, P < 0.05). No difference in IS/AAR between MIR group and c-MB + US group was observed (41.23 ± 8.57% vs 39.65 ± 6.89%, P > 0.05). Treatment with hs-MB and ultrasound caused a significant reduction in IS/AAR as compared with c-MB + US group (25.26 ± 6.44% vs 39.65 ± 6.89%, P < 0.05), representing a 36.3% reduction in infarct size. There was no difference in IS/AAR between hs-MB + US and Na_2_S groups (25.26 ± 6.44% vs 26.82 ± 3.90%, P > 0.05) (Fig. 4F).

### Ultrasound targeted hs-MB destruction preserved left ventricular function

**Figure 5:**
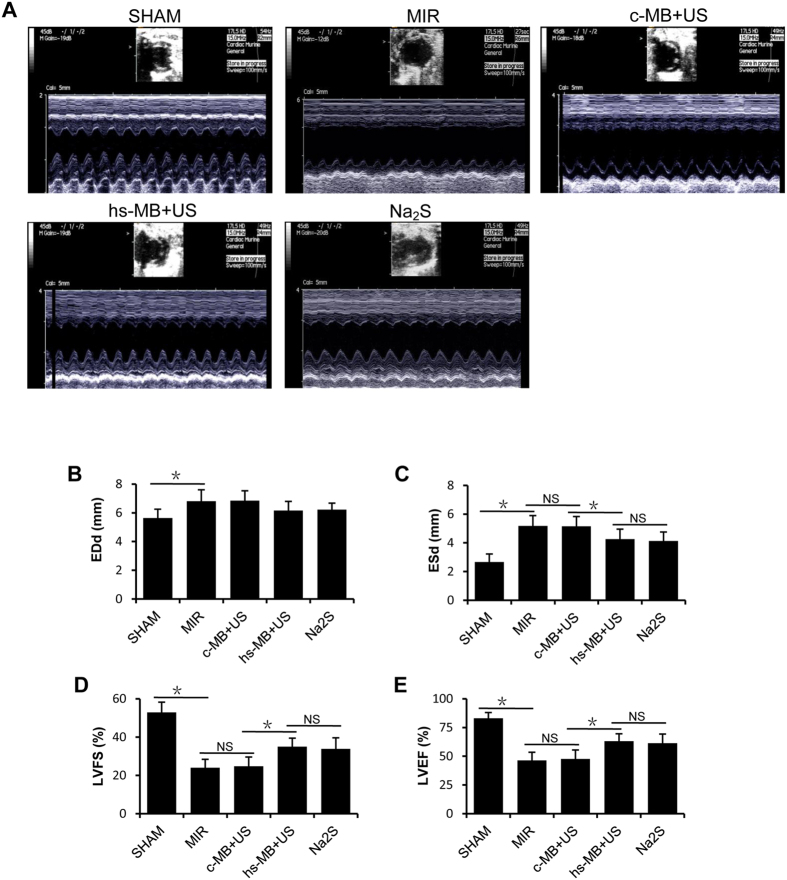
Ultrasound targeted hs-MB destruction preserved left ventricular function. (**A**) The representative echocardiograph images of short-axis view of the left ventricle in M-mode. (**B**) Left ventricular end- diastolic diameter (EDd); (**C**) Left ventricular end-systolic diameter (ESd); (**D**) Left ventricular fractional shortening (LVFS); (**E**) Left ventricular ejection fraction (LVEF). *P < 0.05. MIR indicates myocardial ischemia-reperfusion injury; c-MB, control microbubble; US, ultrasound; hs-MB, microbubble loaded with hydrogen sulfide; NS, not significant.

Left ventricular function was evaluated by echocardiography 24 hour after reperfusion. The representative images show the short-axis view of the left ventricle in M-mode (Fig. 5A). Significant left ventricular dysfunction was observed in rats subjected to MIR compared with the SHAM group. MIR caused an increase in EDd (6.83 ± 0.79 mm vs 5.64 ± 0.62 mm, P < 0.05) and ESd (5.19 ± 0.69 mm vs 2.68 ± 0.56 mm, P < 0.05) and decrease in LVFS (24.01 ± 4.39% vs 52.95 ± 5.28%, P < 0.05) and LVEF (46.47 ± 7.00% vs 83.12 ± 4.92%, P < 0.05). No significant differences in ESd, EDd, LVFS or LVEF were observed between c-MB + US group and MIR group (both P > 0.05). Attenuation of the increased ESd (4.03 ± 0.70 mm vs 5.16 ± 0.68 mm, P < 0.05) but not the EDd (6.16 ± 0.64 mm vs 6.85 ± 0.69 mm, P > 0.05) was observed in hs-MB + US group compared with c-MB + US group. LVFS (34.99 ± 4.48% vs 24.77 ± 4.84%, P < 0.05) and LVEF (63.08 ± 6.47% vs 47.63 ± 7.82%, P < 0.05) were improved in hs-MB + US group compared with c-MB + US group, which shows the preservation of left ventricular systolic function. No significant differences in ESd, EDd, LVFS or LVEF were observed between hs-MB + US group and Na_2_S group (both P > 0.05) (Fig. 5).

### Ultrasound targeted hs-MB destruction alleviated MIR induce apoptosis

**Figure 6:**
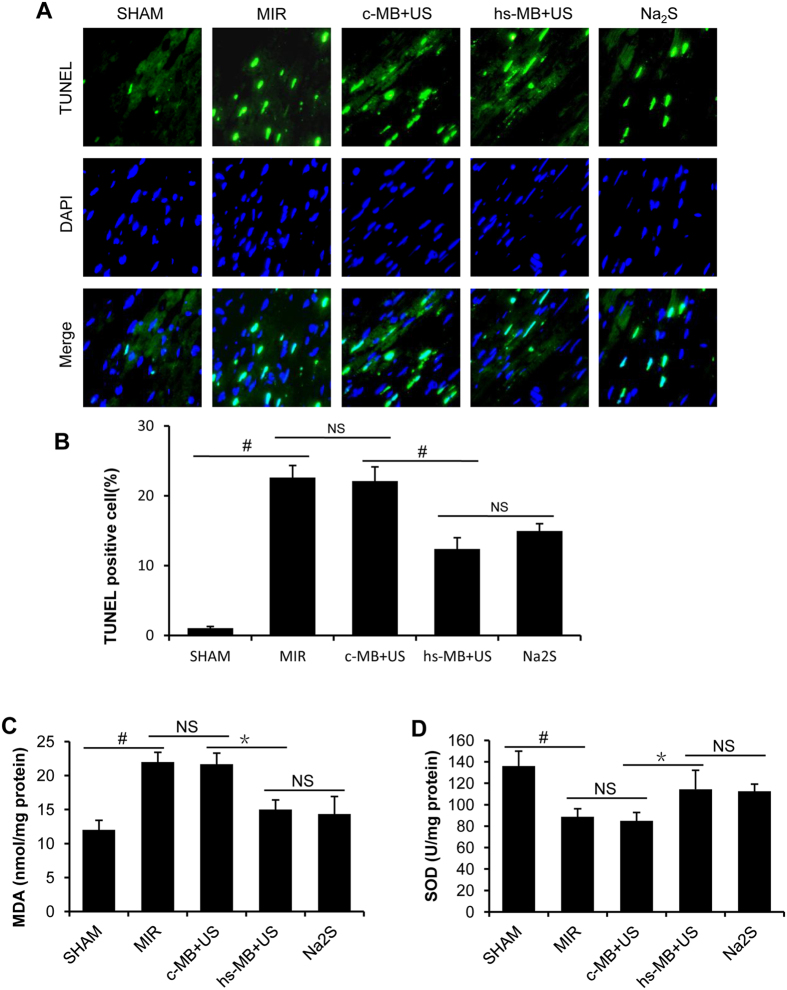
Ultrasound targeted hs-MB destruction alleviated MIR induce apoptosis and oxidative stress. (**A**) Representative pictures of sections stained with TUNEL (×400). Green fluorescence indicated TUNEL-positive apoptotic nuclei; blue fluorescence indicated total cardiomyocyte nuclei. (**B**) Quantification of the TUNEL positive cell. (**C**) MDA level in the myocardium. (**D**) SOD level in the myocardium. *P < 0.05; ^#^P < 0.01. MIR indicates myocardial ischemia-reperfusion injury; c-MB, control microbubble; US, ultrasound; hs-MB, microbubble loaded with hydrogen sulfide; MDA, malondialdehyde; SOD, superoxide dismutase; NS, not significant.

Apoptosis plays a critical role in MIR^24^. TUNEL staining was used to determine if the observed protective effect of hs-MB against MIR injury was associated with decreased apoptosis. Representative photographs of TUNEL staining are shown in Fig. 6A. In response to MIR, total TUNEL positive nuclei were significantly increased compared to the SHAM group (22.63 ± 1.71% vs 1.05 ± 0.24%, P < 0.01). There was no difference in TUNEL labeling nuclei between c-MB + US and MIR group (22.10 ± 2.03% vs 22.63 ± 1.71%, P > 0.05). Significant reduction in TUNEL labeling nuclei was noted in hs-MB + US group compared with c-MB + US group (12.39 ± 1.60% vs 22.10 ± 2.03%, P < 0.01). There was no difference in TUNEL labeling nuclei between hs-MB + US and Na_2_S groups (12.39 ± 1.60% vs 11.95 ± 1.06%, P > 0.05) (Fig. 6B).

### Ultrasound targeted hs-MB destruction attenuated MIR induce oxidative stress

MDA and SOD were determined as a biomarker of pro-oxidative stress and antioxidant respectively. MIR significantly increased the MDA level and reduced the SOD level in myocardium when compared with the SHAM group (P < 0.01). No differences were noted in MDA and SOD between c-MB + US and MIR group (both P > 0.05). There was a marked decreased in MDA (P < 0.05, Fig. 6C) and increased in SOD (P < 0.05, Fig. 6D) in hs-MB + US group in comparison with the c-MB + US group. No differences were observed in MDA and SOD between hs-MB + US and Na_2_S group (both P > 0.05).

### Ultrasound targeted hs-MB destruction had no influence on haemodynamics and respiratory

In order to assess the safety of ultrasound targeted hs-MB destruction, we mornitored blood pressure, heart rate and respiratory rate during treatment. Table 1 showed that no changes in systolic blood pressure, diastolic blood pressure, heart rate and respiratory rate were found among during intervention.

## Discussion

In this study, stable microbubble loaded with H_2_S was prepared with appropriate proportion of H_2_S and C_3_F_8_, which possessed the ability to release H_2_S under ultrasound sonication. Utilizing hs-MB and ultrasound to deliver H_2_S into the myocardium limited the extent of myocardial injury and preserved cardiac function. This cardioprotective effect was associated with alleviation of apoptosis and oxidative stress.

Preparation of the stable hs-MB is the foundation for delivery of H_2_S by UTMD but is quite a challenge. Being a small molecule, H_2_S may efflux from the microbubble shell, resulting in difficult formation and poor stability of hs-MB. It is widely accepted that C_3_F_8_ act as a large molecule internal gas that contributes to microbubble stabilization^25^. Addition of C_3_F_8_ has been shown to enhance the stability of microbubbles loaded with oxygen^26^,^27^ or nitric oxide^28^. We therefore speculated that introducing C_3_F_8_ with H_2_S might increase the stability of hs-MB. However, it should be noted that the more C_3_F_8_ is added the less H_2_S will be encapsulated in the microbubble. To balance the stability and the high H_2_S loading for the microbubble, a mixture of gases at different H_2_S/C_3_F_8_ ratios were used to prepare the hs-MB. As a result, the concentration of hs-MB increased with the increase in content of C_3_F_8_ and reached the point when C_3_F_8_ accounted for more than half of mixture gases. In addition, we found that the concentration of hs-MB decreased sharply within hours at the H_2_S/C_3_F_8_ ratio of 4/0 and 3/1, while there was no change within 3 days at the ratio of 2/2, 1/3 and 0/4. These findings supported the notion that C_3_F_8_ could enhance the concentration and stability of microbubble loaded with H_2_S. C_3_F_8_ enhancement of hs-MB stability may be attributed to the theory that the tendency for H_2_S to diffuse out of the hs-MB is counteracted by the chemical potential gradient of H_2_S to diffuse into the hs-MB diluting the C_3_F_8_ trapped in the microbubble^27^,^29^. Furthermore, our findings indicate that the H_2_S/C_3_F_8_ ratio of 2/2 is an optimal ratio to prepare stable hs-MB with higher H_2_S loading capability, and it offers great promise for delivery of H_2_S.

Delivery of H_2_S to myocardium was achieved using low intensity ultrasound to release encapsulated H_2_S from hs-MB. In the *in vitro* experiments, we found that during infusion of hs-MB, low intensity ultrasound irradiation increased the dissolved H_2_S concentration, which indicated that H_2_S was successfully encapsulated in the hs-MB and its release could be triggered by ultrasound. Next, using myocardial contrast echocardiography, we observed in rats that hs-MB was capable of traveling in the circulation and reaching the myocardium after intravenous infusion. Guided by ultrasound imaging, hs-MB was fragmented in the myocardium using low intensity ultrasound. An intermittent ultrasound delivery mode (3 seconds on and 9 seconds off) enabled sufficient hs-MB to perfuse into the myocardium, which enhanced the effectiveness of H_2_S delivery. Finally, we found that H_2_S concentration was increased in myocardium following hs-MB + US treatment, while not in kidny and liver, suggesting the efficacy of local delivery of H_2_S into myocardium using hs-MB and US. We also found that the H_2_S concentration in lung was increased following hs-MB + US treatment, this may due to the nearby location of lung that easily suffer from ultrasound sonication.

In the present study, we also found that delivery of H_2_S into myocardium by UTMB exhibits cardioprotective effect both in structural and functional terms. In the rodent model of MIR, we directly compared the effectiveness of the H_2_S-loaded MBs with control MB during sonication. Histochemistry showed a significant reduction in infarct size in rats treated with hs-MB and ultrasound. Preservation of cardiac function was evidenced by greater LVEF and LVFS in rats that received hs-MB and ultrasound. The improvements in systolic function could be directly related to the smaller size of the infarct myocardium. These findings are consistent with previous studies of H_2_S as an attractive pharmacological agent for MIR, although the administration strategy they employed was using hydrogen sulfide donors such as NaHS or Na_2_S^5^,^11^,^12^,^30^. The systemic delivery strategy may cause unwanted side effects as mentioned above^18^,^19^,^20^. The delivery strategy for H_2_S we report here is myocardium-specific and has no influence on haemodynamics and respiratory, which may avoid systemic side effects, so has the translation potential for myocardial reperfusion therapy in clinical practice.

We found that apoptosis and oxidative stress were alleviated in rats treated with hs-MB and ultrasound, which may have contributed to the myocardial salvage and improvement of cardiac function. It has been reported that H_2_S induced phosphorylation of glycogen synthase kinase-3β resulted in inhibition of mitochondrial permeability/ transition pore opening, thereby preventing cardiomyocyte apoptosis induced by hypoxia/reoxygenation^31^. Activation of PKC-p44/42-STAT-3 signaling cascade has been reported to reduce apoptotic cell death^10^. The antiapoptotic effect of H_2_S may also relate to the opening of the putative mitochondrial K_ATP_ channels^32^. Our data also showed that ultrasound targeted hs-MB destruction attenuated oxidative stress as evidenced by the change in the SOD and MDA levels. The antioxidant actions of H_2_S are associated with direct scavenging of reactive oxygen species (ROS) or up-regulating antioxidant enzymes. Being a strong reducing agent, H_2_S is able to react with ROS including superoxide anion, hydrogen peroxide, peroxynitrite, and hypochlorite^9^,^33^. H_2_S is capable of activating antioxidant enzymes, such as SOD, to decrease the levels of ROS in cardiomyocytes during ischemia and reperfusion^34^. It is also reported that H_2_S increase Nrf2 nuclear accumulation and subsequent expression of Thioredoxin-1 and Heme Oxygenase-1 to combat oxidative stress^10^.

There are several limitations to our study. First, this study only evaluated the beneficial effect of hs-MB and ultrasound within 24 hours; the long-term effects on myocardial function need to be investigated further. Second, although we found that antioxidant stress and antiapoptotic reaction were associated with cardioprotective effect of ultrasound targeted hs-MB destruction, the specific mechanisms need to be further explored.

In conclusion, we achieved delivery of H_2_S into myocardium using ultrasound and hs-MB prepared with appropriate proportion of H_2_S and C_3_F_8._ UTMD of hs-MB decreases apoptosis and oxidative stress, resulting in reduced myocardial injury and improved cardiac function in a rodent model of MIR. Microbubbles and ultrasound may be a useful method for site-specific delivery of therapeutic gas to avoid unwanted side effects. This novel approach may find clinical use as an adjunct for myocardial reperfusion therapy.

## Methods

### Materials and animals

1,2-dipalmitoyl-sn-glycero-3-phosphocholine (DPPC), 1,2-dipalmitoyl-sn-glycero-3-phosphate sodium salt (DPPA), and 1,2-dipalmitoyl-sn-glycero-3-phosphoethanolamine-N-[methoxy(polyethyleneglycol)-5000] ammonium salt (DPPE-PEG5000) were purchased from Avanti Polar-lipids (Alabaster, AL, USA). H_2_S and C_3_F_8_ were obtained from Foshan Kodi Gas Chemiacal Industry Co., Ltd (Foshan, China). Zinc acetate, N, N- dimethyl-pphenylenediamine sulfate, Na_2_S, 2,3,5-triphenyltetrazolium chloride (TTC) were purchased from Sigma-Aldrich Co (St Louis, MO, USA). *In Situ* Cell Death Detection Kit was purchased from Roche Applied Science (Mannheim, Germany). Propylene glycol, glycerol and ferric trichloride were obtained from Guangdong Guanghua Sci-Tech Co. Ltd (Guangzhou, China). All reagents used in the present study were of analytical grade.

A total of 99 Sprague-Dawley rats (weight 250 to 300 g) were supplied by Southern Medical University (Guangzhou, China). All animal experiments were approved by the Animal Research Committee of the Southern Medical University and were carried out in accordance with the Guide for the Care and Use of Laboratory Animals published by US National Institutes Health (NIH Publication No. 85-23, updated 2011).

### Preparation of hs-MB

DPPA, DPPC, DPPE-PEG5000 (molar ratio of 10:82:8) were dissolved in propylene glycol and heated at 70 °C until the solution was clear. Glycerol and saline were then added into the solution and mixed by rotating to obtain uniform lipid dispersion. The solution was then saturated with H_2_S. Two milliliters of solution was transferred to a 3-mL vial and sealed. Five mixture gases were prepared at five different H_2_S/C_3_F_8_ ratios, including 4/0, 3/1, 2/2, 1/3 and 0/4. The air headspace of each vial was purged with 10 mL of mixture gases (H_2_S/C_3_F_8_) and then activated by a Vial shaker (ZongRay Medical Instrument Company, Hangzhou, China) to prepare the microbubble^35^.

### Characterization of hs-MB

A microscope (OLYMPUS BX51, Olympus Optical, Tokyo, Japan) was used to characterize the morphology of hs-MB. The size distribution and concentration of hs-MB were measured with the Multisizer III Coulter counter (Beckman Coulter Inc., Brea, CA, USA). For stability assessment, the concentrations of the hs-MB were measured at different time points. In order to determine the amount of H_2_S encapsulation, one milliliter of hs-MB was added to a 1-L plastic container and destructed by ultrasound sonication. The containing H_2_S was measured with a portable pump suction H_2_S gas detector (SKY2000-H2S, Shenzhen Yaunte Technology Co., Ltd., Shenzhen, China). The optimal ratio of H_2_S/C_3_F_8_ was figured out and used in the following experiment.

### Ultrasound triggered H_2_S release from hs-MB *in vitro*

Ultrasound triggered H_2_S release from hs-MB was evaluated with the use of a flow system that mimics physiological flow conditions^36^ (Fig. 2A). PBS was infused through the flow system at a constant flow rate of 10 mL/min with a flow pump. hs-MB was infused into the tubing at 100 μl/min. A ultrasound delivery transducer (DCT-700, Shenzhen Well.D Medical Electronic, Shenzhen, China) was placed over the flow system transmitting through a three-centimeter thick tissue mimicking phantom (TMP). The ultrasound with frequency of 1.0 MHz, peak-to-peak pressure of 1.0 MPa and duty cycle of 1.0% at a pulse repetition frequency of 100 Hz was used to fragment the hs-MB. A H_2_S-sensitive polarographic electrode (ISO-H2S-100) connecting to the free radical analyzer TBR4100 (World Precision Instruments, FL, USA) was placed downstream from the flow system for continuous monitoring of dissolved H_2_S concentration. The electrode was calibrated by constructing a standard curve using an EDTA-Na_2_S solution in deoxygenated distilled water according to the manufacturer’s instructions^37^.

### *In vivo* local H_2_S delivery mediated by hs-MB and US

Nine Sprague-Dawley rats were randomly divided into three groups: 1) Control; 2) hs-MB; 3) hs-MB + US. Control group received no treatment. Rats in hs-MB group received 6 × 10^9^/(kg•h) hs-MB via tail vein infusion for 30 minutes. Rats in hs-MB + US group received ultrasound sonication during hs-MB infusion. A ultrasound delivery transducer was placed over the heart to destruct hs-MB with a frequency of 1.0 MHz, peak-to-peak pressure of 1.0 MPa and duty cycle of 1.0% at a pulse repetition frequency of 100 Hz in an intermittent mode of 3 seconds on and 9 seconds off.

The hs-MB perfusion was monitored by an ultrasound imaging transducer as described below. Blood pressure and heart rate were measured using a indirect blood pressure meter (BP2010AUL, Softron Biotechnology Ltd. Beijing, China). Respiratory rate was counted every 5 minutes. The heart, lung, liver and kidney were collected for the the H_2_S measurement following treatment. The tissues were isolated and homogenated in 10 vol of ice-cold PBS, followed by centrifugation for 10 min at 12,000 g. The supernatant was collected and the H_2_S was detected by the free radical analyzer TBR4100 37.

### *In vivo* imaging of ultrasound targeted hs-MB destruction in the myocardium

To observe the behavior of hs-MB and the myocardial perfusion, myocardial contrast echocardiography was performed using an ultrasound system (Sequoia 512, Siemens, Germany) with a imaging transducer(17L5) in the mode of Contrast Pulse Sequencing. The transducer was positioned at the fourth intercostal space to obtain a short-axis image of left ventricle, the depth and gain settings were optimized and held constant. During the infusion of hs-MB, acoustic images were obtained at a frequency of 7 MHz and mechanic index of 0.18 before and during the fragmentation of hs-MB.

### Rodent model of MIR and *In vivo* experimental protocol

Given that the rodent heart needed to be exposed to ultrasound before and during reperfusion, an established closed-chest model of MIR was utilized with minor modification^38^. Rats were fully anesthetized with ketamine (60 mg/kg) and pentobarbital sodium (50 mg/kg), orally intubated, and ventilated. Left thoracotomy was performed in the third intercostal-space and a 5-0 polypropylene suture was placed around the left coronary artery (LCA). Both ends of the suture were threaded through a bead to form a loose snare around the LCA and then exteriorized through the chest wall. The correct position of the LCA ligature was confirmed by observing the paleness of the left heart myocardium after transiently tightened the suture. The bead was left in the chest cavity and the thorax was closed. After the operation, ligation of the LCA was accomplished by tightening the suture until ST elevation appeared on the electrocardiogram. After 30 minutes of ischemia, myocardial reperfusion was achieved by cutting the suture.

The experimental protocols are shown in Fig. 4A. Rats were randomly divided into 5 groups (n = 18 in each group): (1) SHAM: the suture was not tightened after operation and each rat received 6 ml/(kg•h) saline via tail vein injection. (2) MIR: the suture was tightened for 30 minutes and each rat received 6 ml/(kg•h) saline via tail vein injection. (3) c-MB + US: the suture was tightened for 30 minutes and each rat received 6 × 10^9^/(kg·h) control microbubble (prepared with pure C_3_F_8_, c-MB) and ultrasound irradiation. Ultrasound for hs-MB destruction was used as described above. (4) hs-MB + US: the suture was tightened for 30 minutes and rats received 6 × 10^9^/(kg·h) hs-MB and ultrasound irradiation as in group 3. (5) Na_2_S: the suture was tightened for 30 minutes and rats received 100 μg/kg Na_2_S at the time of reperfusion. Treatments were performed five minutes before reperfusion and lasted for 30 minutes. At 4 h of reperfusion, 12 rats in each group were sacrificed for TUNEL staining and MDA and SOD measurement. At 24 h of reperfusion, echocardiography was performed and hearts were harvested for Evans Blue/TTC staining.

### Measurement of MDA and SOD Content in myocardium

Myocardial tissue was obtained and homogenated with appropriate buffer. After centrifugation for 15 min at 3000 g and 4 °C, the supernatant was collected and stored at −70 °C. Superoxide dismutase (SOD) and malondialdehyde (MDA) were measured using commercial assay kits (Nanjing Jianche Bioengineering Institute) according to the instructions of manufacturer.

### Determination of myocardial apoptosis

Myocardial apoptosis was determined by TUNEL staining according to the instructions of the manufacturer. The apoptotic cells were stained green. Nuclei were stained with DAPI in blue. The number of TUNEL positive nuclei and the total number of nuclei per high-powered field were counted using Image-Pro Plus 6.0 (Media Cybernetics, Bethesda, MD, USA) from at least 6 randomly selected fields from the area at risk (AAR) in each section. All measurements were performed in a blinded manner.

### Echocardiographic measurements

Myocardial function was accessed by echocardiography at 24 h after reperfusion using the ultrasound system with a 17L5 transducer (Sequoia 512, Siemens, Germany). Short-axis B-mode images of the left ventricle were acquired at the level of the papillary muscles. Left ventricular end-systolic diameter (ESd) and end-diastolic diameter (EDd) were measured in a blinded manner and left ventricular fractional shortening (LVFS) was calculated as (EDd-ESd)/EDd × 100%. End-diastolic volume (EDv) and end-systolic volume (ESv) were calculated as 7.0 × EDd^3^/(2.4 + EDd) and 7.0 × ESd^3^/(2.4 + ESd) respectively. Left ventricular ejection fraction (LVEF) was calculated as (EDv-ESv)/EDv × 100%^39^.

### Measurement of myocardial infarct size

Myocardial infarct size was measured by Evans Blue/TTC dual staining as previously described^10^. Twenty-four hours after reperfusion, the ligature around the coronary artery was retied before 1 ml of 2% Evans Blue dye was injected into the aorta. The heart was quickly removed and frozen at −20 degrees. The heart was cut into 6 sections and incubated in 1% TTC for 10 min at 37 degrees. Areas not at risk were stained deep blue by Evans Blue. Myocardium at risk but still viable was stained red by TTC. Infarcted myocardium appeared pale after staining. Areas of infarct size (IS) and area at risk (AAR) were measured digitally using Image-Pro Plus 6.0 (Media Cybernetics, Bethesda, MD, USA). Infarct size was calculated as (IS/AAR) × 100% in a blind manner. AAR was composed of red and white area and expressed as (AAR/LV) × 100%.

### Statistical analysis

Statistical analysis was performed with SPSS 19.0 software (SPSS Inc., Chicago, IL, USA). All values are presented as mean ± SD. Comparisons between multiple groups were performed by one-way ANOVA followed by Bonferroni post hoc test. Data of the stability assessment of hs-MB was analyzed using repeated-measures ANOVA. Statistical significance was set at P < 0.05.

## Additional Information

**How to cite this article**: Chen, G. *et al*. Delivery of Hydrogen Sulfide by Ultrasound Targeted Microbubble Destruction Attenuates Myocardial Ischemia-reperfusion Injury. *Sci. Rep*. **6**, 30643; doi: 10.1038/srep30643 (2016).

## Figures and Tables

**Table 1:** No changes in blood pressure, heart rate and respiratory rate when hs-MB administration.

	0 min	5 min	10 min	15 min	20 min	25 min	30 min	35 min	40 min
Systolic blood pressure (mm Hg)									
Control	113 ± 4	111 ± 7	117 ± 3	109 ± 4	113 ± 4	115 ± 4	117 ± 4	116 ± 6	114 ± 4
hs-MB	113 ± 8	114 ± 3	113 ± 7	113 ± 7	114 ± 8	108 ± 10	111 ± 12	112 ± 7	116 ± 6
hs-MB + US	110 ± 14	113 ± 7	109 ± 4	114 ± 5	113 ± 7	111 ± 7	107 ± 15	111 ± 14	111 ± 10
Diastolic blood pressure (mm Hg)									
Control	75 ± 8	77 ± 4	78 ± 9	76 ± 4	73 ± 5	74 ± 3	71 ± 4	71 ± 5	70 ± 5
hs-MB	75 ± 5	70 ± 6	70 ± 5	74 ± 5	70 ± 3	75 ± 7	79 ± 5	71 ± 6	73 ± 4
hs-MB + US	74 ± 8	70 ± 6	73 ± 6	73 ± 5	75 ± 8	76 ± 11	73 ± 10	76 ± 3	75 ± 6
Heart rate (bpm)									
Control	393 ± 12	389 ± 22	387 ± 18	383 ± 18	386 ± 9	3858 ±	388 ± 14	384 ± 21	390 ± 23
hs-MB	381 ± 14	383 ± 7	380 ± 12	389 ± 19	382 ± 18	385 ± 9	395 ± 2	388 ± 14	381 ± 14
hs-MB + US	387 ± 21	369 ± 11	377 ± 23	387 ± 18	377 ± 25	379 ± 18	379 ± 27	374 ± 16	387 ± 17
Respiratory rate (bpm)									
Control	60 ± 4	61 ± 4	61 ± 3	62 ± 3	60 ± 3	61 ± 2	60 ± 7	59 ± 3	60 ± 7
hs-MB	58 ± 6	59 ± 4	60 ± 2	63 ± 7	61 ± 6	60 ± 8	60 ± 7	64 ± 3	59 ± 3
hs-MB + US	63 ± 4	61 ± 6	63 ± 3	63 ± 4	59 ± 4	64 ± 3	62 ± 5	62 ± 9	60 ± 8
